# Effect of decompressive hemicraniectomy in patients with acute middle cerebral artery infarction

**DOI:** 10.3906/sag-2011-66

**Published:** 2021-08-30

**Authors:** Halil İbrahim SÜNER, Anıl TANBUROĞLU, Emre DURDAĞ, Soner ÇİVİ, Aylin GÜNEŞLİ YETİŞKEN, Özgür KARDEŞ, Çağatay ANDİÇ, Kadir TUFAN

**Affiliations:** 1 Department of Neurosurgery, School of Medicine, Başkent University, Adana Turkey; 2 Department of Neurology, School of Medicine, Başkent University, Adana Turkey; 3 Department of Radiology, School of Medicine, Başkent University, Adana Turkey

**Keywords:** Decompressive hemicraniectomy, stroke, middle cerebral artery

## Abstract

**Background/aim:**

We aimed to determine in which cases this procedure may be more effective based on the data of patients who underwent decompressive hemicraniectomy (DHC).

**Material and methods:**

Overall, 47 patients who underwent DHC due to acute middle cerebral artery (MCA) infarction between January 2014 and january 2019 were retrospectively investigated. These patients were divided into two groups: those who died after DHC (Group A) and those who survived DHC (Group B). The groups were compared in terms of various parameters. We investigated whether the patient’s modified Rankin scale (mRS) status changed depending on age (> 60 and < 60 years).

**Results:**

The median age of all patients was 65 (37–80) years; groups A and B had median ages of 66.5 (37–80) and 61 (44–79) years (p = 0.111), respectively; 55.3% patients were male. The elapsed times until hospitalization after the onset of symptoms were 4.5 and 3 h in groups A and B, respectively (p = 0.014). The median GCS score at the time of admission was 7 (5–12) and 10 (8–14) in groups A and B, respectively (p = 0.0001). At the time of admission, 63.3% patients in group A had anisocoria, whereas no patient in group B had anisocoria (p = 0.0001). In postoperative period, 40% patients in group A and all patients in group B received AC/AA treatment. The survival of patients aged < 60 and > 60 years who underwent DHC for MCA infraction was 61.5% and 26.5%, respectively (p = 0,041). The median mRS of patients < 60 and > 60 years were 4 (1–6) and 6 (1–6), respectively (p = 0.018).

**Conclusion:**

Age, DHC timing, and elapsed time until hospitalization or access to treatment directly affect the functional outcome and survival in MCA-infarcted patients who underwent DHC. In patients in whom the medical treatment fails, early DHC administration will increase survival without waiting for neurological worsening once herniation is detected radiologically.

## 1. Introduction

Decompressive hemicraniectomy (DHC) is an auxiliary intervention to treat high intracranial pressure (ICP) caused by acute stroke and traumatic brain injuries. The nutrition and oxygenation of the brain are disturbed in acute stroke where cerebral blood perfusion (CBP) decreases [1,2]. Morbidity or death caused by herniation and cerebral edema can be prevented because of DHC.

The treatment of severe cerebral infarction and large cerebral edema caused by acute stroke is one of the most controversial neurovascular phenomena. Such severe cerebral infarctions occur in 1%–10% of all supratentorial infarctions [3]. Cerebral edema due to acute stroke may lead to herniation and eventually mortality or morbidity despite antiaggregant thrombolytic therapy or mechanical thrombectomy administered during the first 24 h [4,5]. Therefore, early DHC is recommended to reduce ICP and achieve better functional results [4]. Ipsilateral DHC after an acute stroke caused by a thrombus of the middle cerebral artery (MCA) was first reported in 1956 [6]. DHC can help with brain decompression, prevent herniation and play a life-saving role by increasing CBP. Most patients are discharged with a severe disability although the risk of mortality in patients with MCA infarction decreases with DHC [7].

In this study, we examined the patients who underwent DHC due to progressive cerebral edema because they did not respond to aggressive medical treatment after MCA infarction, were not suitable for thrombectomy, or because of failed thrombectomy. We divided the patients with MCA infarction who survived and died despite DHC administration into two groups. We investigated the factors affecting survival and functional recovery based on the different characteristics of the groups.

## 2. Materials and methods

Patients who underwent DHC at our clinic between January 2014 and January 2019 due to increased ICP caused by different etiologies and related neurological worsening were retrospectively investigated. Data of the patient were collected using hospital data and archiving system. Patients who were unable to visit the hospital for regular follow-up were called by phone. We included only cases of acute MCA infarction in which DHC was used to reduce ICP elevation. The patients included in the study were admitted to the Neurology Intensive Care Unit (NICU) after their emergency service admissions. They were followed up at the NICU after DHC procedure. Patients with MCA infarction who underwent DHC were divided into two groups: deceased patients (group A) and surviving patients (group B). The outcomes and post discharge data of patients in group B were collected from their files and through phone calls.

### 2.1. Parameters

The two groups were compared in terms of age, sex, systemic diseases; prior use of anticoagulants and antiaggregants (AC/AA) treatments, admission complaints, elapsed time until hospitalization after the onset of symptoms; Glasgow Coma Scale (GCS) score at admission, presence of anisocoria at the time of admission, application of tissue plasminogen activator (tPA); the success of thrombectomy during the preoperative period, preoperative and postoperative AC/AA treatments, DHC decision-making criteria (clinical deterioration, radiological deterioration or both); the presence of constraints and/or blood on presurgical brain computed tomography (CT), presence of anisocoria at the time of DHC, elapsed time from admission to DHC (DHC time); the side that DHC was applied, performance of duraplasty and hematoma evacuation during the surgical procedure and presence of blood on the postoperative CT and at the follow-up period. All patients were followed by preoperative and postoperative CT. Group B was evaluated according to the discharge modified Rankin scale (mRS) (Table 1) [8,9], outpatient follow-up period and cranioplasty time. We also investigated if the mRS score and outcomes of patients varied depending on age (> 60 and < 60 years) and the side of pathology (right and left MCA infarction).

**Table 1:** Modified Rankin scale (mRS) (24.22).

Score	Description
0	No symptoms at all
1st	No significant disability despite symptoms, able to perform usual duties and activities
2nd	Slight disability, unable to perform all previous activities but able to look after own affairs without assistance
3rd	Moderate disability, requiring some help but able to walk without assistance
4th	Moderately severe disability, unable to walk without assistance and unable to attend own needs without assistance
5th	Severe disability, bedridden, incontinent and requiring constant nursing care and attention
6th	Dead

### 2.2. Data analysis

Data were analyzed using the SPSS package software program (Version 17.0, SPSS Inc., Chicago, IL, USA). Normality was analyzed with the Kolmogorov–Smirnov and Shapiro–Wilk tests and histograms for each continuous variable. All numerical data were expressed in median values (minimum-maximum), and categorical variables were described as proportions. The categorical variables between the groups were analyzed with the Chi square or Fisher’s exact tests. The groups were compared using the Mann–Whitney U test for the non-normally distributed data. A p value <0.05 was considered significant.

## 3. Results

### 3.1. Participants

A total of 67 patients underwent DHC for increased ICP due to different etiologies and related neurological deterioration. Forty-seven patients were included who underwent DHC due to ICP elevation resulting from malignant MCA infarction, which could not be avoided despite medical and interventional treatment. Those who underwent DHC for tumors, trauma and etiologies other than acute stroke were not included in the study. The patients were divided into two groups: group A (deceased patients, n = 30) and group B (surviving patients, n = 17).

### 3.2. Age, sex, comorbidities, medicines and complaints

The median age of all patients was 65 (37–80) years. The median ages of the A and B groups were 66 (37–80) and 61 (44–79) years, respectively; it was insignificant (p = 0.111). The majority of patients were male (55.3%), and there was no significant difference between the groups in terms of sex (group A: male, 53.3%; female, 46.7% and group B: male, 58.8%; female, 41.2%; Table 2). The most common reason for admission was hemiparesis/hemiplegia (45.9%) followed by impaired consciousness (37.93%) and speech impairment (16.09%).

**Table 2:** Comparison of the groups in terms of age, sex, history of systemic disease, anticoagulant or antiaggregant use, arrival time to the hospital, and surgical timing parameters.

	Age(years)(p = 0.111)(median(min-max))	Sex(p = 0.768)(%)	Comorbidities (%)HT(p = 0.704)CAD(p = 0.500)DM(p = 0.094)AC/AA(p = 0.912)	Arrival time to the hospital (hour) (p = 0.014)(median(min-max))	DHC Timing(hour)(p = 0.991)(median(min-max))
Group A(n = 30)	66.5(37–80)	M: 53.3F: 46.7	HT: 83.3CAD: 23.3DM: 36.7AC/AA: 46	4.5(1–120)	42(4–480)
Group B(n = 17)	61(44–79)	M: 58.8F: 41.2	HT: 76.5CAD: 35.3DM: 11.8AC/AA: 52.94	3(1–8)	48 (6–144)

*AC/AA: Anticoagulant and Antiaggregant, CAD: Coronary artery disease, DHC: Decompressive Hemicraniectomy, DM: Diabetes Mellitus, HT: Hypertension, F:Female M:Male.

The most common systemic diseases were hypertension (HT) in 80.9%, coronary artery disease (CAD) in 27.7%, diabetes mellitus (DM) in 27.7% and atrial fibrillation (AF) in 25.53%. The groups’ HT rates were similar, and they were not significant (p = 0.704), 88.2% of group B did not have DM (p = 0.094). Additionally, 23 (48.93%) patients were using at least one of the AC/AA medications such as dabigatran etexilate, apixaban, clopidogrel, warfarin sodium and acetylsalicylic acid (ASA) due to previous systemic or recovered diseases. Hence, there was no difference between the groups (p = 0.912) (Table 2).

### 3.3. Arrival and surgery times

Overall, 74.5% of patients were referred from other healthcare centers to our clinic. The arrival time of patients to our center since the onset of symptoms was 4.5 h in Group A and 3 h in Group B. This parameter was significant between the groups (p = 0.014). The median surgery time after the onset of symptoms was 44 h (4–480) in all patients. This parameter was not significant, and both groups were operated in 42 and 48 h on average, respectively (p = 0.991, Table 2).

### 3.4. Neurological status

All patients were evaluated based on their GCS scores and the presence of anisocoria at the time of admission to our hospital. The median GCS score at the time of admission was 8 (5–14) and 40.8% of the patients had anisocoria. The median GCS score at the time of admission was 7 (5–12) and 10 (8–14) in groups A and B, respectively and it was significant (p = 0.0001). At the time of admission, no one in group B had anisocoria, while 63.3% of the patients in group A did. At the time of surgery, anisocoria was present in 83.3% and 47.1% of patients in groups A and B, respectively (Table 3). Anisocoria assessments at the times of hospital admission and surgical treatment were significant between the groups (p = 0.0001 and p = 0.018, respectively).

**Table 3:** Comparison of the groups in terms of anisocoria and GCS at the first examination, preoperative anisocoria, side of pathology, and CT findings.

	Anisocoria(at first examination)(p = 0.0001)(%)	GCS(at first examination)(p = 0.0001)(median(min-max))	Anisocoria(Preoperative)(p = 0.018)(%)	Side of pathology(p = 1.000)(%)	Preoperative CT(B and H)(p = 0.544, p = 0.059)(%)
Group A(n = 30)	63.3	7 (5–12)	83.3	Right: 56.7Left: 43.3	B: 73.3H: 96.7
Group B(n = 17)	0	10(8–14)	47.1	Right: 52.9Left: 47.1	B: 41.2H: 88.2

*B: Blood, H: Herniation, GCS: Glasgow coma scale.

### 3.5. Radiology

**Figure 1:**
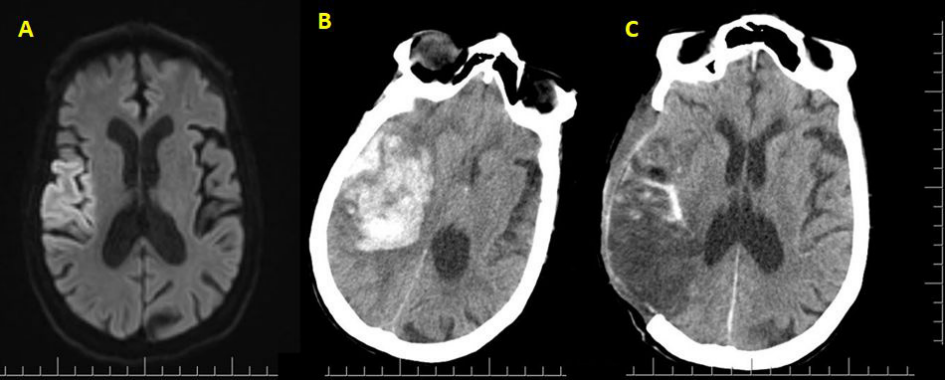
A patient with right MCA infarction. A: Early MRI findings. B: Intracerebral hematoma and subfalcin herniation on CT 24 h after admission. C: CT findings after DHC and hematoma evacuation. *DHC: Decompressive hemicraniectomy, MCA: Middle cerebral artery, CT: Computed tomography, MRI: Magnetic resonance image.

All patients underwent CT examinations upon admission to our hospital and NICU. Right MCA infarction was observed in 55.3% of the patients, and both groups had similar rates (56.7% and 52.9%, respectively) (p = 1.000). Radiological herniation findings were observed in 96.3% (n = 44) of the patients. The patients had uncal and subfalcine herniation or both (96,7% and 88.2% of patients in groups A and B, respectively, p = 0.059). Intracerebral hematoma or subarachnoid hemorrhage were observed in 61.7% (n = 29) of patients. Although it was not significant, 73.3% and 41.2% of patients in groups A and B had intracerebral hematoma or subarachnoid hemorrhage on CT, respectively (p = 0.544) (Table 3). Six patients (5 in Group A and 1 in Group B) were operated on with DHC due to intracerebral hematoma (Figures 1A, 1B). They were treated with surgical evacuation (Figure 1C).

### 3.6. tPA and thrombectomy

tPA was used in 38.3% of patients during the preoperative period, and it was not different between the groups (groups A: 33.3% and B: 47.1%, p = 0.371). Moreover, 46.7% and 5.9% of patients in groups A and B, respectively underwent thrombectomy with endovascular intervention during the preoperative period and it was significant (p = 0.004, Table 4).

**Table 4:** Comparison of the groups in terms of preoperative tPA, thrombectomy, and preoperative and postoperative AC/AA treatments.

	tPA(p = 0.371)(%)	Thrombectomy(p = 0.004)(%)	PreoperativeAC/AA treatment(p = 0.363)(%)	PostoperativeAC/AA treatment(p = 0.023)(%)
Group A(n = 30)	33.3	46.7	66.7	60
Group B(n = 17)	47.1	5.9	2.4	100

*AC/AA: Anticoagulant and Antiaggregant, tPA: Tissue plasminogen activator.

### 3.7. Anticoagulant and antiaggregant treatment

Patients were examined in terms of their preoperative and postoperative AC/AA treatments in the NICU. The treatment regimens provided during the preoperative period did not differ between the groups (p = 0.363) and AC/AA treatment was administered to all patients in group B during the postoperative period. Approximately 40% of group A did not receive this treatment due to increased risk of postoperative bleeding or existing bleeding, which was observed in both groups, and it was significant (p = 0.023, Table 4). The most preferred treatment during the postoperative period was the combined administration of low-molecular-weight heparin and ASA to 40% and 58.5% of patients in groups A and B, respectively.

### 3.8. Surgery

The criteria for the choice of the surgical treatment were evaluated. DHC was assessed based on the clinical and radiological statuses of the patients. Surgical treatment was deemed appropriate for the 14.9% of patients for neurological deterioration (sudden decrease of 2 or more points in GCS) and in 31.9% due to worsening in the radiological findings, and there was no significant difference between the groups. DHC was performed as standard on the side of pathology in all patients with an average size of 15 × 15 cm to cover the frontal, parietal and temporal lobes, and the dura was opened. The median operative time in all patients was 75 (60–150) min and there was no significant difference (group A: 75 (60–150) min and group B: 90 (60–120) min). Six patients (12.8%) had hematoma evacuation. Further, 70.6% and 56.7% of patients in groups B and A, respectively did not undergo duraplasty (with allograft or autograft). Three patients (two patients in group A and one in group B; 6.38%) were re-operated due to wound complications during the postoperative follow-up. 

### 3.9. The follow-up

**Figure 2:**
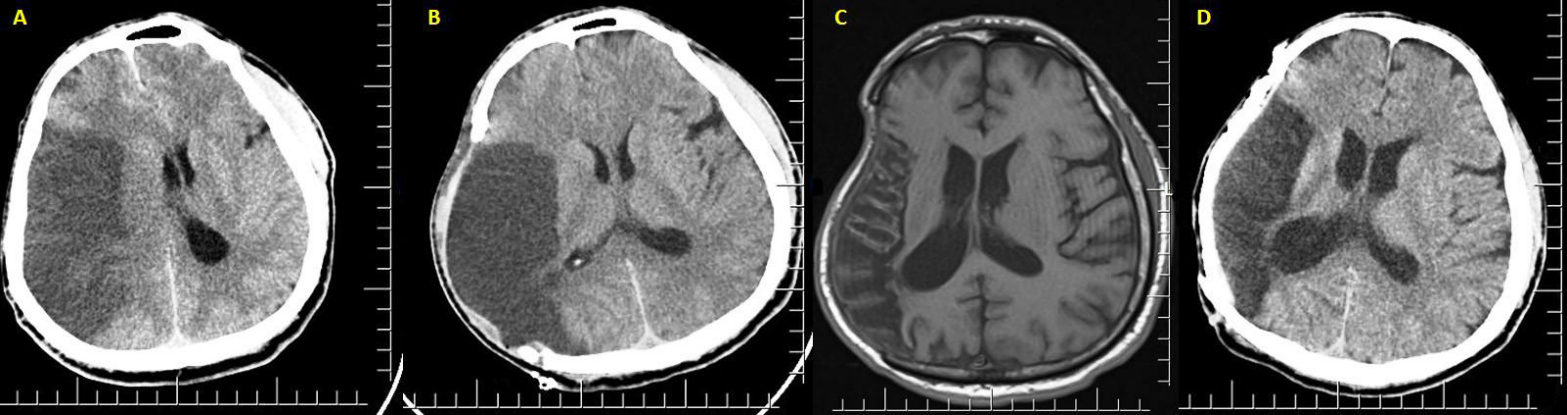
A 45-year-old patient underwent DHC due to right MCA infarction. Upon arrival at the hospital, his GCS was 14 and he had right MCA infarction, shift and edema were observed on CT (A). Herniation improved after DHC (B). Encephalomalastic area was observed on MRI 6 months later (C), and cranioplasty was performed (D). *DHC: Decompressive hemicraniectomy, MCA: Middle cerebral artery, GCS: Glascow coma scale, CT: Computed tomography, MRI: Magnetic resonance imagine.

The medical treatments were continued in NICU during the follow-up. The median postoperative follow-up time of patients in group A who died despite the medical and surgical treatments targeting malignant MCA infarction was 10.5 (1–72) days. The median discharge time of patients in group B was 22 (10–63) days. The mean GCS score of patients in group B during discharge was 11.12 ± 1,867. Fourteen patients were followed up for an average period of 10.6 months. Three patients, one of whom did not visit the hospital after the operation and two died due to heart attack and pneumonia, were not followed. Three clinically suitable patients underwent cranioplasty (Figures 2A, 2B, 2C, 2D). Of the patients who underwent DHC in group B, those with right-side pathology had a better median mRS score (median: 2, min-max:1–4). The median mRS score of those with right MCA infarction in group B at the time of discharge was significant (p = 0.001).

### 3.10. Outcome

The median age of the groups was not significant. The median age of group B was 61 (44–79) years, and this group included younger patients. All patients were categorized as aged > 60 and < 60 years to investigate the effect of age on patients with MCA infarction and the ones who underwent DHC. The survival rate of MCA infarcted patients aged <60 years was 61.5%, while it was 26.5% in those aged > 60 years and it was significant (p = 0.041). The median mRS of patients < 60 and > 60 years were 4 (1–6) and 6 (1–6), respectively. The difference in mRS between the age groups was significant (p = 0.018, Table 5). Patients who underwent DHC were assessed in separate groups of left and right MCA infarctions. The survival rate of patients with right MCA infarction and who underwent DHC was 34.6% and their median mRS score was 6 (1–6). The survival rate of patients with left MCA infarction was 38.09%, and their median mRS score was 6 (3–6). The effect of pathological side differences on the outcome and mRS was not significant (p = 0.232).

**Table 5:** mRS and survival status of the patients by age.

Age groups(years)	n	Survival(p = 0.041)(%)	mRS(p = 0.018)(Median (min-max))
< 60	13	61.5	4 (1–6)
> 60	34	26.5	6 (1–6)

*mRS: Modified Rankin scale.

## 4. Discussion 

Large hemispheric infarctions are observed in 1%–10% of the patients with supratentorial infarction [10]. Life-threatening brain edema is usually seen between the second and fifth day after the onset of stroke and the prognosis for these patients is poor despite maximum intensive care treatment [11,12]. The mortality rate of the cases was 70%–80% in intensive care-based prospective series [4,12]. So, the term “malignant” MCA infarction is used for large cerebral infarctions that do not respond to medical treatments such as sedation, hyperventilation, steroid, barbiturate, glycerol and mannitol and various conservative treatment strategies aimed at reducing brain edema and ICP. In such cases where other treatments are inadequate, DHC is a surgical treatment option that reduces mortality by reducing ICP, stopping herniation and increasing CBP. Preoperative ICP monitoring can help decide the necessity of DHC. Postoperative ICP monitoring is also useful to decide if additional treatment is required.

DHC is generally performed using a one-sided approach on the infarction side with which a bone flap of 15 × 15 cm is removed [13]. The dura is incised and exposed or wide duraplasty is performed. The brain expands from the skull outward. In decompressive surgery, which involves only bone removal, ICP decreases nearly by 15%, while this decrease after DHC can increase to 70% if the dura is also exposed [13,14]. We did not perform duraplasty or dura exposure, and the dura of all patients were left open after DHC in the present study. In terms of functional outcome and mortality, there was no difference between duraplasty or dura exposure via durotomy.

Many researchers have focused on this life-saving surgical option in the last 20 years. They often drew attention to DHC time and the effectiveness of the procedure through age and functional outcomes. Although there are different opinions, the younger patients have better results than those aged ≥ 60 years [15]. However, the analysis of a large Japanese database in which DHC patients aged > 60 years constituted 80% of the population, showed no age-related differences [16]. The American Heart Association and the American Stroke Association have recommended DHC within the first 48 h, especially in cases of stroke in people aged < 60 years [17]. The Neurocritical Care Society and German Society for Neuro Intensive Care and Emergency Medicine recommended DHC within 24–48 h regardless of age [18]. Similar to our study, they stated that patients > 60 years may be more likely to have a serious disability. Of the patients who underwent DHC in our study, the median age of the surviving patients was 61 (44–79) years and it was 66.5 (37–80) in the deceased. Similar to the literature, all patients aged > 60 and < 60 years were re-evaluated, and young age had a significant impact on survival. Performing DHC in stroke cases of young age assisted in achieving better functional recovery.

Cardiac diseases lead to a stroke, which is possibly caused by a cardio embolic stroke. Similar to our study, the most common diseases in patients with MCA infarction were HT, DM, hyperlipidemia and AF and these diseases and smoking were important risk factors for stroke [5]. Comorbidity was directly associated with mortality and functional outcome [19]. In the present study, 80.9% of patients had HT, 27.7% CAD, 27.7% DM and 25.53% AF and our data were in line with the literature.

Low GCS score, the poor state of consciousness and anisocoria are indications for herniation. In a study by Huh et al., patients with high preoperative GCS scores had lower mortality rates and better functional outcomes [20]. Similarly, anisocoria was directly related to a bad outcome. In our series, the absence of anisocoria before surgery and the high GCS score at the time of admission increased the chances of survival. Therefore, DHC should be performed before pupil dilatation. Low GCS is a risk factor for unassisted life after discharge in patients with stroke [21].

Some studies on acute stroke therapy investigate the effect of time and urgent intervention that can limit cerebral damage [22]. We reviewed the elapsed time until hospitalization and access to medical treatment after the onset of symptoms. The group of patients surviving DHC had been hospitalized earlier, and early and rapid treatment in ischemic stroke was of great importance.

Many previous DHC analyses recommend early surgery [17,18]. We can associate it with the increased survival owing to DHC performed before clinical manifestations of herniation as in our study. DHC may be more effective for functional recovery and survival if it’s performed within the first 24 h following the onset of the disease [1]. Schwab et al. reported a mortality rate of 16% in the group operated in the first 21 h and a mortality rate of 34.4% in the group operated in the first 39 h on average. The rate of uncal herniation in the latter was 75%, while it was 13% in the first [23]. Dasenbrock et al. analyzed a large national database of 1300 patients who underwent DHC [24]. They found that 56% of patients underwent DHC within 48 h, and only the group operated 72 h after the onset of stroke had worse results. However, there was a significant relationship between the time, herniation and outcome. Therefore, DHC is the most important temporal factor before the development of herniation. This clinical condition can be explained by increased cerebral edema caused by deterioration and ICP that causes impaired cerebral perfusion in the non-ischemic parenchyma as part of a cascade, known as secondary brain damage [25]. The median elapsed time until surgery in patients in our study was 44 (4**–**480) h; however, there was no significant difference between the survivors and those deceased. The positive effect of early hospital admission on survival was significant. Accordingly, the time of admission and time of surgery is the most important prognostic factors in patients with MCA infarction. 

Avoiding postoperative AC/AA treatment due to the risk of hemorrhagic transformation or growing hematoma in patients who underwent DHC and who routinely use AC/AA medications may give rise to other risks. All patients who undergo DHC should be administered AC/AA treatment recommended by neurologists and neurosurgeons to protect them from fatal complications such as deep vein thrombosis, pulmonary thromboembolism, acute coronary syndrome and recurrent stroke. AC/AA treatment should be provided at the neurosurgeons’ discretion. Subcutaneous low-molecular-weight heparin may be administered to prevent deep vein thrombosis despite the hemorrhagic transformation on CT [18]. In our study, patients who received AC/AA treatment during the post-operative period had a lower mortality rate. Rapid reperfusion of ischemic penumbra within 3 h from the onset of symptoms with thrombolysis is a proven method of treatment n acute stroke. Although more patients were administered tPA in the surviving group, it was not significant. In the literature, the rate of unsuccessful thrombectomy in patients with acute stroke was 17% [26]. In our study, a higher number of failed thrombectomy attempts were noted in the deceased group and this was significant. This could be associated with the development of vasospasm after endovascular treatment or the continuation of ICP elevation due to lack of recanalization. In their 690-case series, Park et al. reported that 136 patients (19.7%) had recanalization failure and most of patients with failed endovascular intervention had poor outcomes [27].

Some guidelines are often referred to in the decision of DHC. In the 2018 guide of the American Heart Association and the American Stroke Association, DHC is recommended for all patients with MCA infarction who have neurological worsening within 48 h despite medical treatment and regardless of age [28]. In our study, surgery decisions were made by different surgeons based on only radiological deterioration, only neurological deterioration or both. None of them had any effect on mortality and functional recovery.

DHC for malignant stroke has a significant preventive effect on mortality [29]. In our study, the numbers of patients with left and right MCA infarctions were almost similar, and there was no difference in mortality rates. Although it was not statistically different, patients with right MCA infarction had better mean scores of mRS in terms of functional outcome. This provided a different view. In addition to its life-saving effect, DHC allowed patients with MCA infarction in the dominant hemisphere to live indirectly with moderate/severe disability. However, this is a separate topic of discussion where some ethical issues can be raised. Therefore, having precise information about the relevant data is crucial for patients in the decision-making process and such data should be shared with patients’ relatives while deciding on DHC. It can be difficult to decide who is a candidate for early or urgent surgery and whether surgical delay might be beneficial until clear evidence is found. It might be even more difficult to determine if the patient will have acceptable disability and quality of life different from the predicted based on preoperative estimates [29]. 

Despite being relatively simple, DHC is a demanding surgical procedure since it has significant complications. Complications of DHC increased in patients with advanced age, taking ASA or other anticoagulants [30]. The development of postoperative hematoma or increase in existing hemorrhage due to the routine previous use of AC/AA treatment or the administration of this treatment in NICU during the preoperative period can be considered as the most important complications. Surgical site problems and CSF fistula can be considered as other minor complications due to surgery. In our study, no patients developed hematoma requiring surgical treatment after DHC. However, three patients had surgical wound’s problems.

## 5. Conclusion

To conclude, age, DHC time and elapsed time until hospitalization or access to treatment are directly related to the functional outcome and survival. To increase survival, patients with MCA infarction should be administered medical treatment and DHC once herniation is detected radiologically without waiting for neurological deterioration (anisocoria, low GCS score). Other life-threatening complications can be avoided with proper AC/AA treatment after DHC.

## Limitations

Cranioplasty could not be performed in some patients and were not followed in the long-term due to their city of residence or economic condition.
